# Mechanical alignment tolerance of a cruciate-retaining knee prosthesis under gait loading—A finite element analysis

**DOI:** 10.3389/fbioe.2023.1148914

**Published:** 2023-03-30

**Authors:** Yichao Luan, Huizhi Wang, Chaohua Fang, Min Zhang, Junwei Li, Ningze Zhang, Bolun Liu, Jian Su, Cheng-Kung Cheng

**Affiliations:** ^1^ Key Laboratory of Biomechanics and Mechanobiology, Ministry of Education, Beijing Advanced Innovation Center for Biomedical Engineering, School of Biological Science and Medical Engineering, Beihang University, Beijing, China; ^2^ School of Biomedical Engineering, Shanghai Jiao Tong University, Engineering Research Center of Digital Medicine, Ministry of Education, Shanghai, China; ^3^ Department of Sport Medicine, Ningbo No 6 Hospital, Ningbo, China

**Keywords:** alignment tolerance, knee prosthesis, gait loading, stress, contact pressure, finite element analysis

## Abstract

Component alignment is one of the most crucial factors affecting total knee arthroplasty’s clinical outcome and survival. This study aimed to investigate how coronal, sagittal, and transverse malalignment affects the mechanical behavior of the tibial insert and to determine a suitable alignment tolerance on the coronal, sagittal, and transverse planes. A finite element model of a cruciate-retaining knee prosthesis was assembled with different joint alignments (−10°, −7°, −5°, −3°, 0°, 3°, 5°, 7°, 10°) to assess the effect of malalignment under gait loading. The results showed that varus or valgus, extension, internal rotation, and excessive external rotation malalignments increased the maximum Von Mises stress and contact pressure on the tibial insert. The mechanical alignment tolerance of the studied prosthesis on the coronal, sagittal, and transverse planes was 3° varus to 3° valgus, 0°–10° flexion, and 0°–5° external rotation, respectively. This study suggests that each prosthesis should include a tolerance range for the joint alignment angle on the three planes, which may be used during surgical planning.

## 1 Introduction

Total knee arthroplasty (TKA) is the most effective treatment for severe osteoarthritis. With successive developments over the past several decades, this treatment method often provides positive clinical outcomes and prolonged survival (Hamel et al., 2008; Madry, 2022). However, nearly 20% of patients report dissatisfaction with the postoperative outcome because of pain and restricted function (Bourne et al., 2010). Malalignment of the components is presented as one of the primary factors affecting postoperative outcomes and can lead to revision (Thiele et al., 2015; Luan et al., 2022). Good alignment after TKA leads to faster rehabilitation and better joint functionality (Longstaff et al., 2009; Abdel et al., 2018).

Component alignment on the three planes (coronal, sagittal, and transverse planes) is considered during preoperative planning. Owing to differences in design features and material properties between knee prostheses, recommendations for prosthesis alignment are provided as specific angles by the manufacturer. Surgeons consider it acceptable when the alignment error is within ±3°; otherwise, it is regarded as an outlier (Abdel et al., 2014). Over 16% of component alignments have been reported as outliers in TKA procedures performed using conventional instrumentation (Luan et al., 2022). Computer navigation systems, personal-specific instrumentation (PSI), and robotic surgeries have recently been introduced to improve alignment accuracy and reduce outliers. Still, a study on the accuracy of insertions had shown that 22% of lower limb alignments were misaligned by greater than 3° when the procedure was performed through robotic surgery (Deckey et al., 2021). Similarly, high rates of outliers have also been found in surgeries using PSI and computer navigation (Victor et al., 2014; Swamy et al., 2022). These results demonstrate the difficulty with accurately inserting knee prostheses according to the manufacturer’s instructions in all TKA procedures. Clinical surgeons should accept a more reasonable alignment tolerance as standard practice when planning TKA surgery.

In hospital orthopedic departments, prostheses used in TKA undergo a comprehensive and rigorous assessment by relevant departments before being considered for use. Nevertheless, adverse events are still frequently reported after implantation. While many complications can be linked to component alignment and surgical practices, some may also be traced back to challenges with the pre-clinical testing of orthopedic implants (Cheng et al., 2019); the discrepancies in test conditions and clinical employment are the possible reasons. ISO 14243 (ISO. 14243-1, 2009; ISO. 14243-3, 2014) and ASTM F3141 (ASTM. F3141-17a, 2017) are the most wildly used standards for the pre-clinical wear testing of knee prostheses; In contrast, both standards require the prosthesis to be installed in the standard alignment position, the normal alignment position is rarely replaced in TKA procedures. Previous studies have shown that component alignment affects the kinematics of the knee joint, including the femoral anteroposterior translation and tibial rotation (Shih et al., 2020; Wang et al., 2021; Fang et al., 2022). Malignment can also produce higher stress and contact pressure on the tibial insert, which may increase wear and lead to premature failure (Rostoker and Galante, 1979; Liau et al., 1999; Liau et al., 2002; Cheng et al., 2003; Koh et al., 2020). Therefore, it is not sufficient to only assess the standard alignment position during pre-clinical testing, various alignments based on clinical feedback should be evaluated, with consideration also given to increasing the alignment tolerance.

In recent studies, Gheorghiu et al. found that the 3°varus alignment increased the contact pressure of the tibial liner more than the neutral alignment (Gheorghiu et al., 2021). Tang et al. investigate pressure distribution in the knee at different lower limb alignments with diverse positions of femoral and tibial components by cadaver experiments and finite analysis; the results showed that the peak pressure on the medial or lateral side of the tibial liner was determined by the mechanical axis (Tang et al., 2022). However, these two studies only analyzed the influence of the different alignments under the static compression load. Suh et al. investigated the biomechanical effect of varus and valgus malalignment on the tibial liner under stance-phase gait cycle loading conditions; they suggested greater total contact pressure in the varus alignment than in the valgus, with a more marked difference on the medial side (Suh et al., 2017). Besides, Kang et al. examined the influence of femoral malrotation on the contact pressure of the tibial liner. The results showed that the contact pressure on the medial side of the polyethylene insert increased with internal femoral malrotation and decreased with external femoral malrotation. In contrast, there was an opposite trend in the lateral side of the liner (Kang et al., 2016). Still, the influence of the whole gait cycle needs further study, as well as the stress of the tibial liner, which is related to the pitting and delamination of the polyethylene (Pascaud et al., 1997; Kurtz et al., 2000). The purpose of this study is to investigate how component malalignment on the coronal, sagittal, and transverse planes affect the mechanical behavior of the tibial insert under the gait loading and to determine the mechanical alignment tolerance of the prosthesis on the three planes, which could be applied to the surgeons with proper pre-surgical plans.

## 2 Materials and methods

### 2.1 Model reconstruction

**FIGURE 1:**
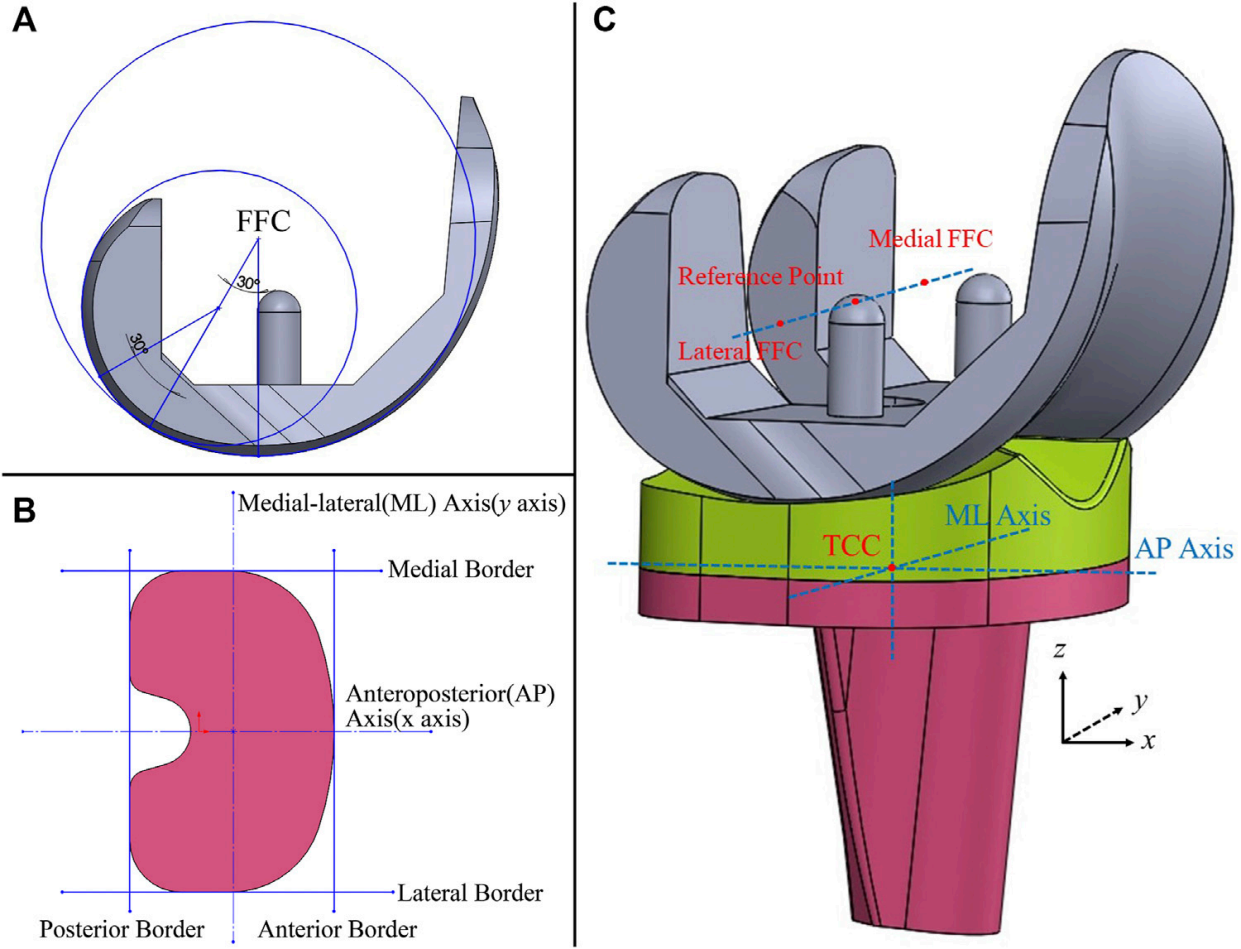
3D model of the knee prosthesis **(A)**: Definition of the femoral flexion center (FFC); **(B)** anteroposterior and medial-lateral axes of the tibial component; **(C)** the coordinate system of the model, TCC, tibial component center).

A three-dimensional model of a cruciate-retaining prosthesis (Size D, NexGen CR-Flex, Zimmer Inc., Warsaw, IN, United States) was reconstructed in the SolidWorks 2016 (Dassault Systèmes SolidWorks Corporation, United States) by reverse engineering a prosthesis retrieved from a patient. The model consisted of three components: a femoral component, a tibial component, and a polyethylene liner. The femoral and tibial components’ elastic modulus and Poisson ratio were 220 GPa and 0.3, respectively. The liner was modeled as ultrahigh molecular weight polyethylene (Elasticity modulus = 495 MPa, Poisson’s ratio = 0.46, yield stress = 20.2 MPa) (Gomoll et al., 2002). The model’s coordinate system was determined according to ISO 14243-3:2014 (ISO. 14243-3, 2014). On the femoral component, the medial and lateral femoral flexion center (FFC) was defined as the intersection point of the normal lines of contact points when the femoral component was flexed to 30° and 60°. The mid-point of the medial and lateral FFCs was set as the reference point of the femoral component. The flexion-extension (FE) axis was defined by a line connecting the medial and lateral FFCs (Figure 1A). In the coordinate system, the anteroposterior (AP) axis (*x*-axis) of the tibial component was a central line of the most lateral and medial borders. A line drawn perpendicular to the AP axis was set as the medial-lateral (ML) axis (*y*-axis) (Figure 1B), and the intersection point of the AP and ML axes was the tibial component center (TCC) located on its upper surface. The *z*-axis was defined by a line perpendicular to the upper surface passing through TCC (Figure 1C).

Mesh convergence testing of the liner was performed in Abaqus 6.14 (Dassault Systèmes Simulia Inc., France) under a static compressive load of 3600N. The contact area, maximum contact pressure, and Von Mises stress are shown in Table 1 as the number of elements in the liner model was increased. Convergence was assumed when the change in their parameters was less than 5%. The results showed that convergence was achieved using 49527 C3D8 elements.

**TABLE 1:** Mesh convergence analysis.

Mesh size (mm)	Mesh number	Mesh type	Contact area (mm^2^)	Contact pressure (MPa)	Mises stress (MPa)
2	14072	C3D8	226.95	27.88	16.84
1.5	32304	C3D8	221.59	27.95	17.69
1	49527	C3D8	220.54	28.04	17.85
0.75	64515	C3D8	219.86	28.35	17.96

### 2.2 Boundary and loading conditions

The femoral and tibial components were set as rigid bodies, and the liner was deformable. The femoral and tibial components were assembled by placing the most distal points of the femoral condyles on the lowest points of the polyethylene tibial liner. The liner was bonded to the tibial component, and the femoral component was set to contact the tibial liner with a friction coefficient of 0.04 (Fitzpatrick et al., 2016). The femoral component was only permitted to flex around the flexion-extension axis, while the tibial component was restricted in the flexion-extension but permitted to move in all other degrees of freedom (Wang et al., 2019; Wang et al., 2021; Fang et al., 2022).

**FIGURE 2:**
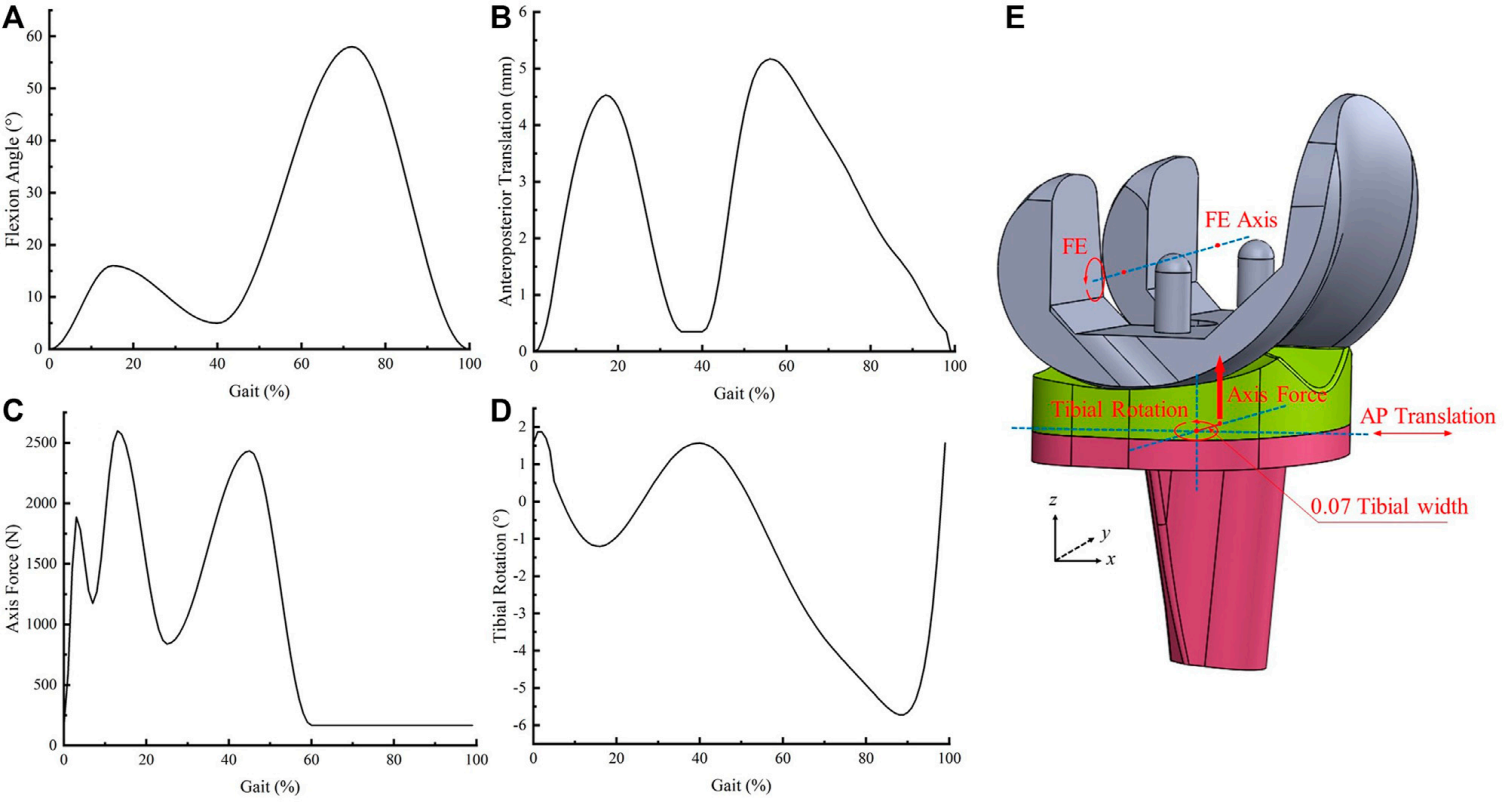
Input curves and loading conditions **(A)**: flexion angle; **(B)** anteroposterior translation, **(C)** axial force, **(D)** tibial rotation, **(E)** schematic diagram of loading conditions).

Dynamic simulations of one gait cycle were performed in Abaqus 6.14 with the analyzed time of one second according to ISO 141243-3. Four curves were loaded into the model to simulate the gait cycle, including flexion-extension, anteroposterior translation, axial force, and tibial rotation (ISO. 14243-3, 2014) (Figures 2A–D). The FE was loaded at the femoral reference point around the FE axis. The axial force was loaded along the *z*-axis but offset to the medial side by a distance of 0.07 times the width of the tibial component. The AP translation and tibial rotation were loaded on the tibial component center (ISO. 14243-3, 2014) (Figure 2E). The tibial component and liner moved anteriorly and rotated internally when the AP translation was greater than 0.

### 2.3 Model validation

The model was validated by applying a compressive load of 3600N through the center of the femoral component, and the contact area and pressure of the tibial insert under different femoral flexion angles (0°, 30°, 60°, 90°) were calculated and compared with experimental results using the same prosthesis. The boundary and loading conditions on the model were applied according to the experimental study (Shiramizu et al., 2009). The trend of the maximum Von Mises stress on the tibial liner throughout the gait cycle was also compared with results from a similar simulation study (Bauer et al., 2021) which used the same loading conditions according to ISO 14243-3:2014.

### 2.4 Simulation of Prosthesis Malalignment

**FIGURE 3:**
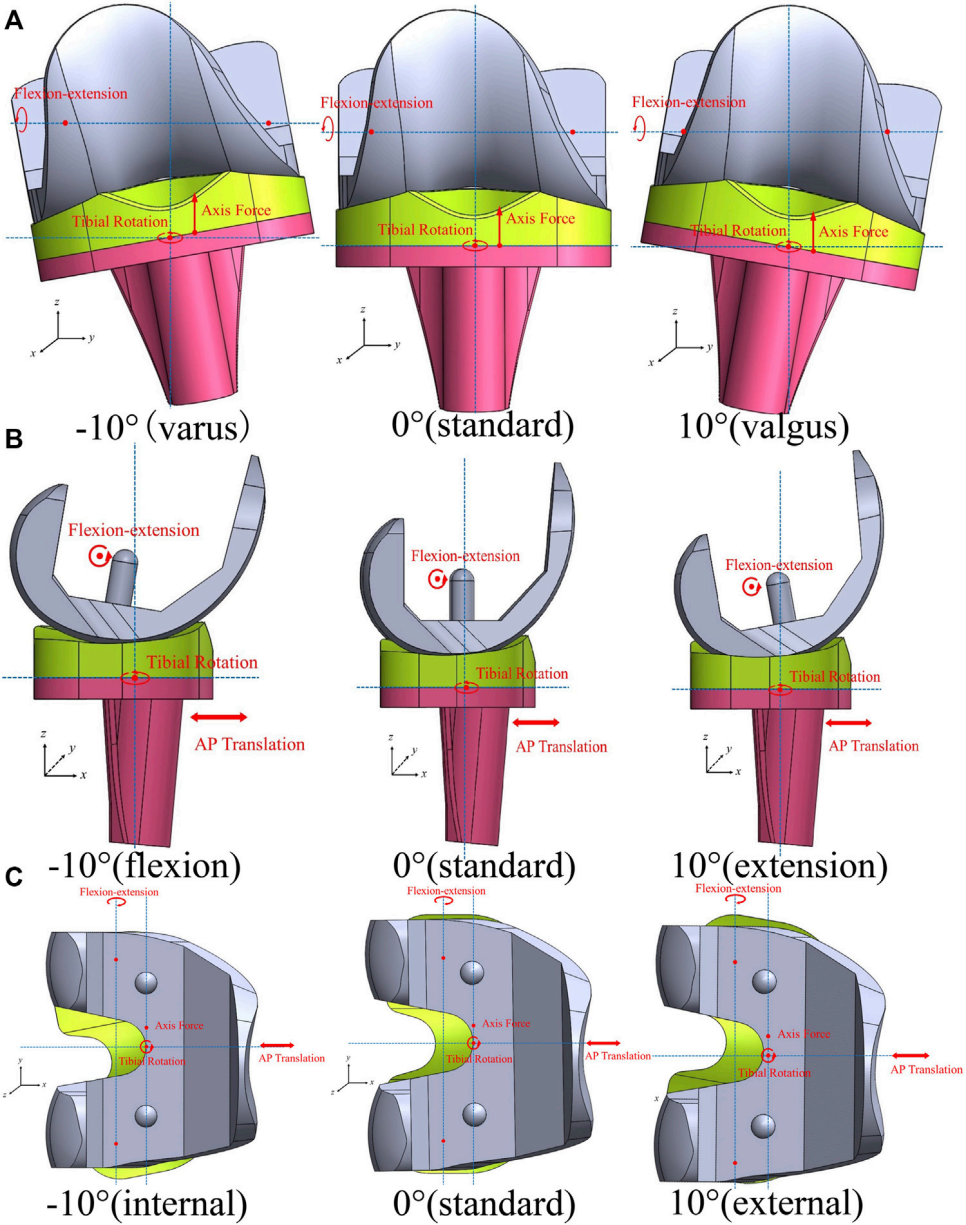
Malalignment of the knee prosthesis and coordinate systems of loading conditions **(A)**: coronal plane; **(B)** sagittal plane; **(C)** transverse plane).

Joint malalignment was simulated using different initial alignment angles of −10°, −7°, −5°, −3°, 3°, 5°, 7°, and 10°, which were established separately on the coronal, sagittal, and transverse planes. The coordinate systems of the four loading curves did not change. On the coronal plane, the joint line of the knee prosthesis was rotated around the AP axis to simulate the malalignment. Therefore, the flexion-extension axis and the internal-external rotation axis were not parallel or perpendicular to the joint line (Schroeder et al., 2022) (Figure 3A). The femoral component was flexed or extended along the FE axis before the gait loading to indicate the malalignment on the sagittal plane (Figure 3B). The tibial component was rotated internally or externally around the *z*-axis when assembling the model to simulate rotational malalignment (Figure 3C). Twenty-five models were simulated, and the Von Mises stress and contact pressure were collected at a 1% increment of the gait loading. The tolerance limits for mechanical alignment were determined when the maximum Von Mises stress reached the yield stress.

## 3 Results

### 3.1 Model validation

**FIGURE 4:**
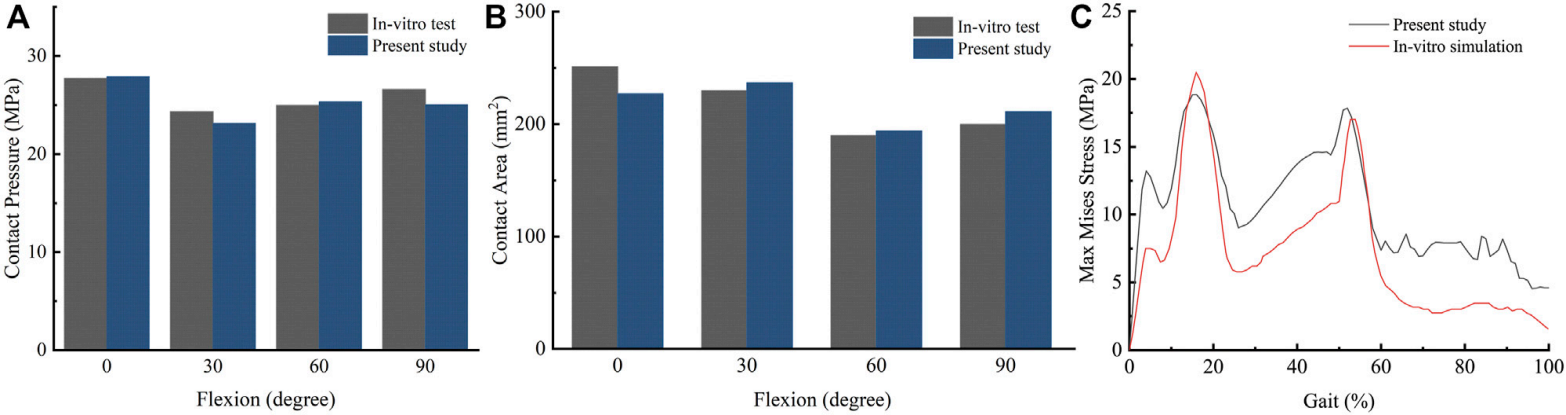
Comparison between previous studies and the present study **(A)**: contact pressure at different flexion angles; **(B)** contact area at different flexion angles; **(C)** maximum Von Mises stress during gait loading).

In the static compressive simulation, when the knee was flexed to at 0°, 30°, 60°, and 90°, the contact pressure was 27.88, 23.13, 25.35, and 25.03 MPa and the contact area was 226.9, 236.81, 193.72, and 210.97 mm^2^ respectively, which were close to reported results from an *in-vitro* study (Shiramizu et al., 2009) (Figures 4A, B). Figure 4C compares the maximum Von Mises stress between Bauer’s study (Bauer et al., 2021) and this study during the gait loading, demonstrating a similar trend between the two studies, although there were some differences in values.

### 3.2 Influence of coronal malalignment on the liner

**FIGURE 5:**
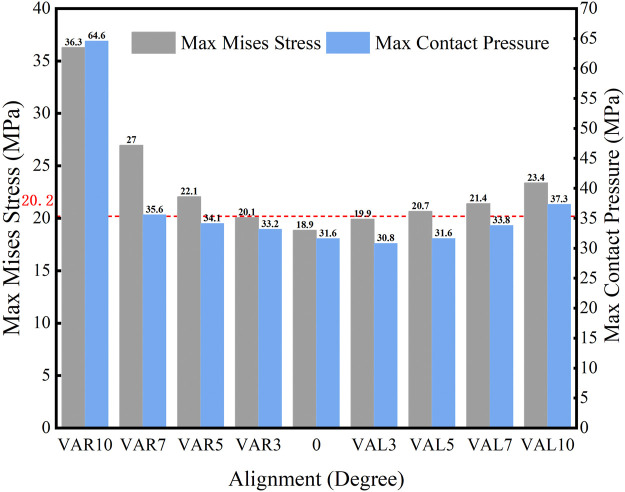
Maximum Von Mises stress and contact pressure with different coronal malalignments (VAR: varus malalignment; VAL: valgus malalignment; red line: yield stress = 20.2 MPa).

The maximum Von Mises stress and contact pressure on the tibial liner with different coronal malalignments are shown in Figure 5. The maximum Von Mises stress and contact pressure with a neutral alignment (0°) were 18.9 MPa and 31.6 MPa, respectively. Both varus and valgus malalignment increased the stress and contact pressure on the tibial liner. When the malalignment was maintained within 3° of varus and valgus, the maximum Von Mises stress during gait loading was less than the yield stress of the liner (20.2 MPa). The range of mechanical tolerance on the coronal plane was 3° varus to 3° valgus.

**FIGURE 6:**
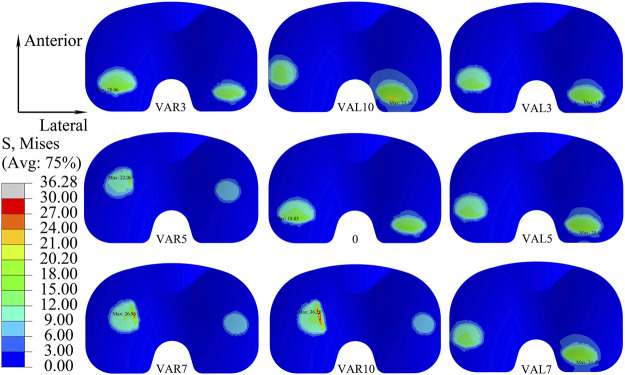
Distribution of maximum Von Mises stress on the liner with different coronal malalignments of the knee prosthesis (VAR: varus malalignment; VAL: valgus malalignment).

The distribution of maximum Von Mises stress on the liner with different coronal malalignments of the knee prosthesis is described in Figure 6. Higher stress was found on the lateral side with the valgus malalignment, while it was identified on the medial side with the varus malalignment.

### 3.3 Influence of sagittal malalignment on the liner

**FIGURE 7:**
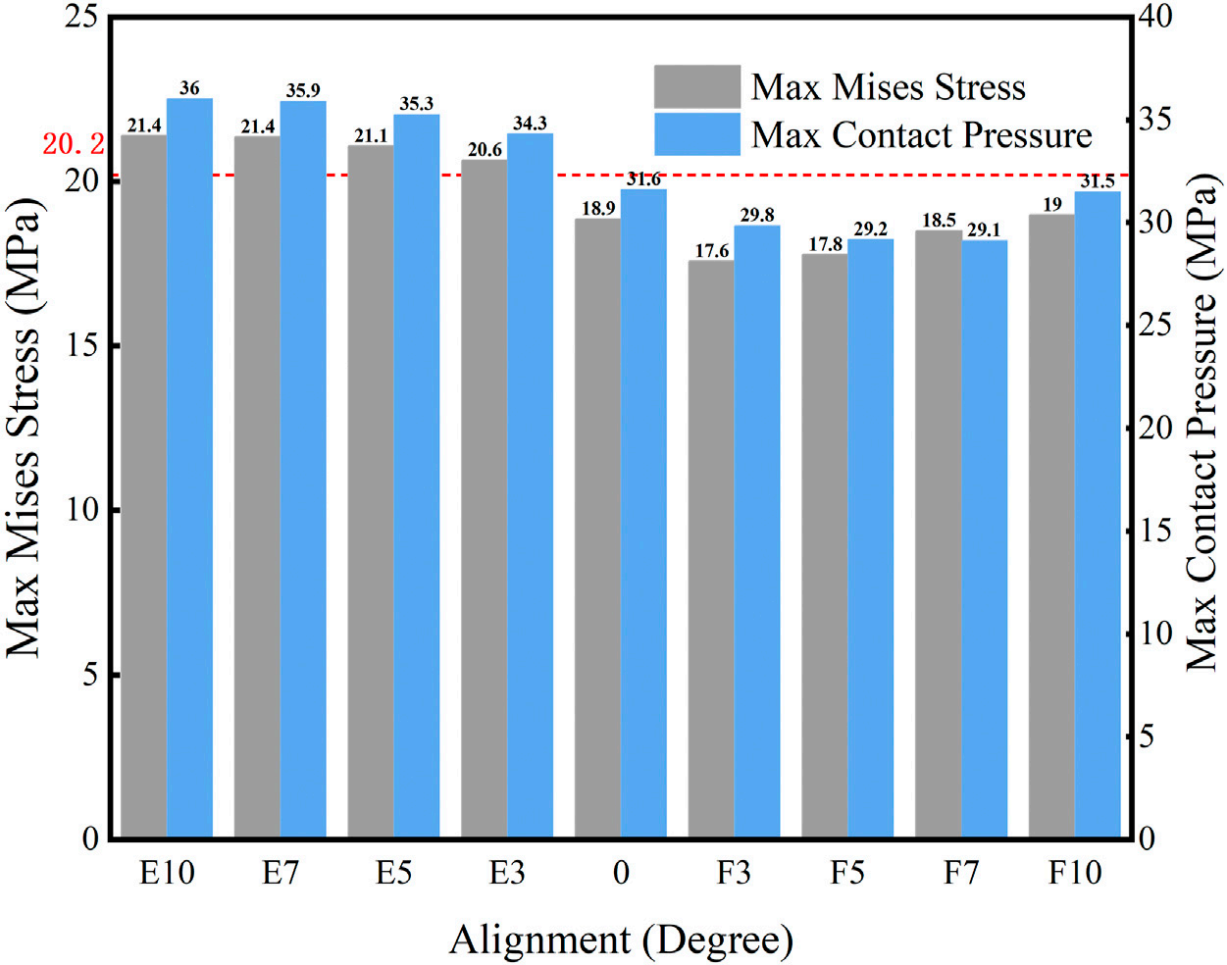
Maximum Von Mises stress and contact pressure with different sagittal malalignments of the knee prosthesis (E: extension malalignment; F: flexion malalignment; red line: yield stress = 20.2 MPa).

The maximum Von Mises stress and contact pressure on the liner when the knee prosthesis was malaligned in the sagittal plane are shown in Figure 7. Higher stress and contact pressure were recorded when the femoral component was in hyper-extension alignment. However, flexed malalignment of less than 10° resulted in stress and contact pressure below the yield stress of 20.2 MPa. The maximum stress on the tibial liner when the knee was extended to 3° was 20.6 MPa, which was slightly above the yield stress, and the stress further increased with larger extensions. Therefore, it is suggested to implant the femoral component with a neutral or moderate flexion alignment and avoid aligning the knee in extension.

**FIGURE 8:**
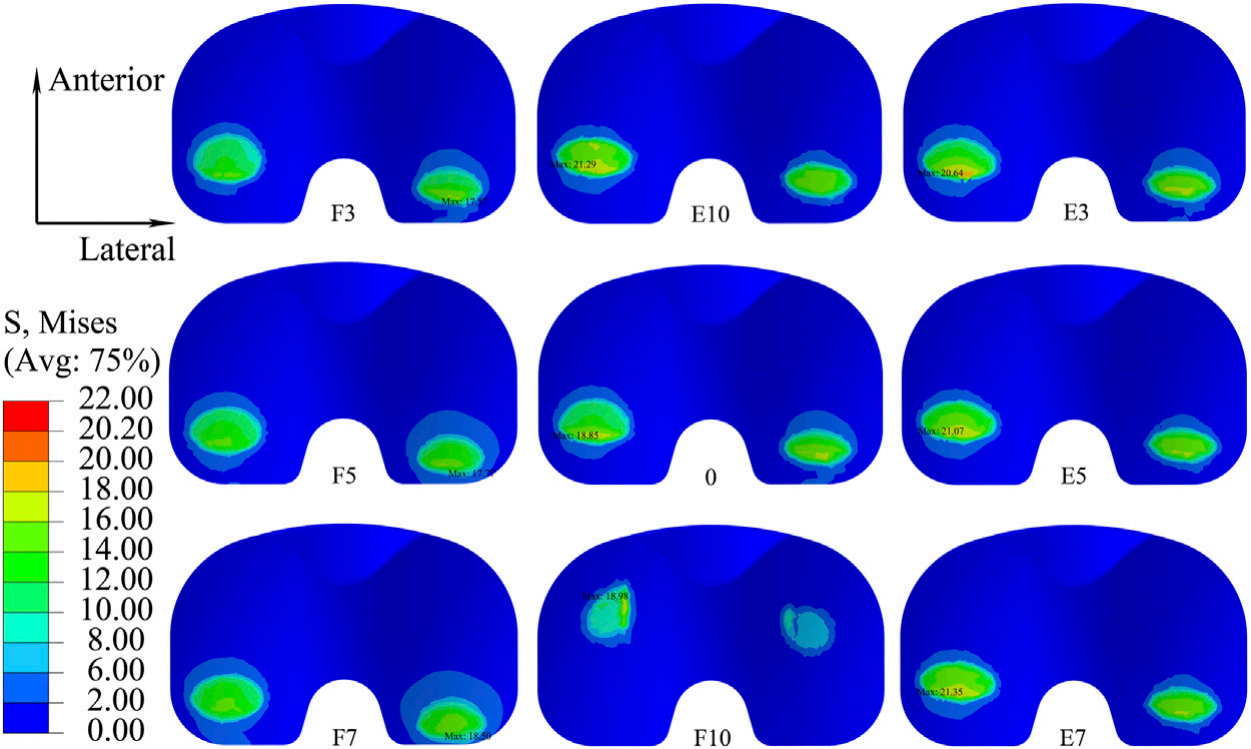
Distribution of maximum Von Mises stress on the liner with different sagittal malalignments of the knee prosthesis (E, extension malalignment; F, flexion malalignment).

The distribution of the Von Mises stress on the liner with different sagittal malalignments of the knee prosthesis is presented in Figure 8. The maximum stress was located on the posterior side of the liner, and the degree of sagittal malalignment did not have an obvious impact on the stress distribution, except for with 10° flexion malalignment, which caused the peak stress on the liner to move anteriorly.

### 3.4 Influence of transverse malalignment on the liner

**FIGURE 9:**
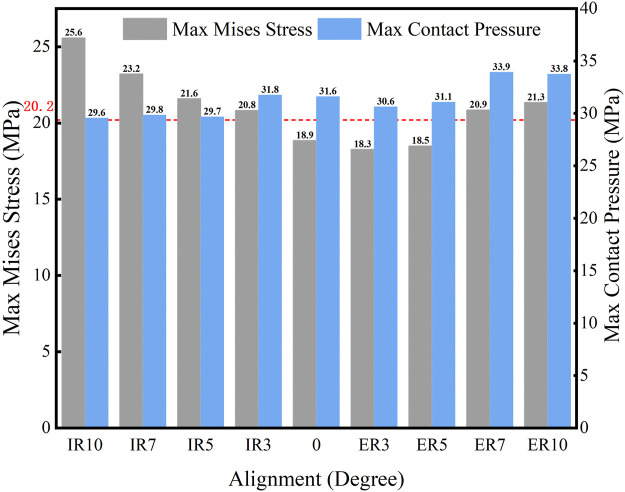
Maximum Von Mises stress and contact pressure with different transverse malalignments (IR: internal rotation malalignment; ER: external rotation malalignment; red line: yield stress = 20.2 MPa).

The maximum Von Mises stress and contact pressure on the tibial liner when the joint was malaligned in the transverse plane are shown in Figure 9. Moderate external rotation (less than 5°) of the tibial component produced less stress and contact pressure than the neutral alignment. However, higher stress and contact pressure were present when the joint was internally or externally rotated by over 5°. When the knee was malaligned by 3° and 5° of external rotation, the stress on the liner was 18.3 MPa and 18.5 MPa, respectively, less than the yield stress. For all other simulated malalignments in the transverse plane, the maximum stress on the liner exceeded its yielding stress of 20.2 MPa. Therefore, the tolerance on the transverse plane was 0°–5° external rotation.

**FIGURE 10:**
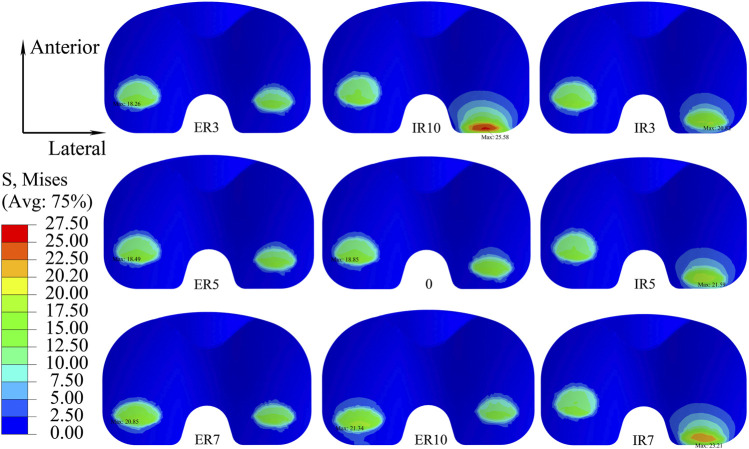
Distribution of maximum Von Mises stress with different transverse malalignments of the knee prosthesis (IR: internal rotation malalignment; ER: external rotation malalignment).

Figure 10 shows the distribution of maximum Von Mises stress on the liner when the knee prosthesis was malaligned by various angles in the transverse plane. The maximum stress under gait loading was located on the posterior side of the liner when in neutral alignment. The point of maximum stress tended to move to a posterior-lateral location for internal malrotation of the knee. It was positioned in a posteromedial location when the knee was externally malrotated.

## 4 Discussion

The main finding of this study is that the initial component alignment plays an important role in the mechanical behavior of knee prostheses. Malalignment of the knee prosthesis may increase the stress and contact pressure during gait loading. For the Nexgen CR knee prosthesis used in this study, the mechanical alignment tolerance on the coronal, sagittal, and transverse plane was 3° varus to 3° valgus, 0°–10° flexion, and 0°–5° external rotation, respectively. When the knee was aligned outside of these tolerance ranges, the stress on the tibial liner exceeded the yield stress of the material, which might increase the risk of wear and lead to premature failure of the liner.

While malalignment of a knee prosthesis affects the overall mechanical behavior of the knee joint, the tibial liner made is typically the most affected and complications associated with the liner are one of the primary reasons for TKA revision (Liau et al., 1999; Liau et al., 2002; Cheng et al., 2003; Cerquiglini et al., 2018). Varus and valgus malalignment on the coronal plane increased the stress and contact pressure on the liner during gait loading. The malalignment changed the force line and distribution of force, which would generate an extra medial or lateral force on the tibial liner. For the simulation, the axis of the axial force was placed medially at a distance of 0.07 times the width of the tibial component according to ISO 14243-3:2014. Hence, the stress and contact pressure on the liner were higher with varus malalignment than with valgus malalignment. This finding is similar to a related study (Suh et al., 2017), where the higher contact pressure and stress influenced the prosthesis’s clinical outcomes and survival rate. Kuroda (Kuroda et al., 2019) reported that valgus alignment resulted in lower clinical scores than neutral and varus. Moreover, Fang (Fang et al., 2009) found that the survival rate was highest when the coronal alignment between the femoral and tibial anatomical axis was 3°–7°.

Flexion malalignment of the femoral component caused the contact position on the tibial liner to move slightly anterior during the gait loading. The smaller curvature surface around the contact position increased the contact area and decreased the contact pressure and stress compared with the larger curvature surface. On the contrary, extension malalignment led to a posterior contact between the femoral component and tibial liner, increasing contact pressure and stress. Therefore, differences in the curvature of the contact face on different liners would affect the allowable alignment tolerance of the knee prosthesis. Manufacturers must provide specific alignment tolerances based on the prosthetic design characteristic. On the sagittal plane, extension malalignment of the femoral component has more of a negative impact on the clinical outcome than flexion malalignment. It has been demonstrated as one of the risk factors leading to failure after TKA (Luan et al., 2022). In this study, internal malrotation and excessive external malrotation on the transverse plane caused higher stress and contact pressure on the tibial liner than the prosthesis with a neutral alignment. This was likely due to the shift in contact position and area, as with the sagittal malalignment. The tibial component was rotated internally by nearly 6° during the gait loading, and the internal malrotation would increase the tibial rotation during gait. The AP translation of the femoral condyles caused a more posterior-lateral contact between the femoral and tibial components because of the internal malrotation. The smaller contact area resulted in higher contact pressure and stress. Tibial internal rotation and excessive external rotation have been implicated in increasing the risk of failure of knee prostheses, and it is suggested to avoid such alignments during TKA procedures (Kim et al., 2014; Luan et al., 2022).

Mechanical alignment is considered the gold standard for total knee arthroplasty and one of the most widely used methods. Positioning the knee prosthesis in a neutral position on the coronal plane within the range of 3° varus to 3° valgus is considered an acceptable alignment tolerance by most clinical surgeons (Bonner et al., 2011). However, recent studies have shown that a neutral alignment did not improve the clinical outcome or survival rate, and a tolerance of 3° varus to 3° valgus could not be considered a “safe zone” with modern personalized alignment strategies, especially for an updated prosthesis (Tibbo et al., 2021; Schelker et al., 2022). A possible reason is considerable differences in the design of contemporary knee prostheses, which can affect the mechanical behavior and wear performance (Asseln et al., 2021; Zhang et al., 2021; Su et al., 2022). Although advanced techniques, like personalized specific instrumentation, navigation systems, and robotic surgeries, have improved surgical precision and alignment accuracy, malalignment is still a common complication. The single angle for component alignment recommended by the manufacturer is insufficient and cannot be applied to all TKA procedures. Therefore, a specific range for the alignment tolerance of every prosthesis must be available to clinical surgeons when planning the surgical approach.

There are some limitations to this study. First, only one prosthesis was assessed, and the mechanical alignment tolerances are only applicable for this design since it is suggested that the alignment tolerance is unique for each prosthesis. Second, the 3D model did not incorporate bone and soft tissues because this study aims to assess the mechanical behavior of the tibial insert under gait loading according to ISO 14243-3, so the effects of bone and soft tissue were not considered. Similarly, this study only assessed gait loading. Activities like up and down stairs and squatting may be considered in future work. Finally, this study did not evaluate the effect of combined malalignment on different planes and needed further investigation.

## 5 Conclusion

The malalignment angles of the knee prosthesis on the coronal, sagittal, and transverse planes influence the stress and contact pressure of the tibial liner under gait loading. To meet the yield condition of polyethylene and avoid plastic deformation of the liner, the alignment tolerance of the studied prosthesis on the coronal, sagittal, and transverse planes was 3° varus to 3° valgus, 0°–10° flexion, and 0°–5° external rotation, respectively. Manufacturers are suggested to provide a tolerance for the joint alignment angle on the three planes to give more information for surgeons to make proper pre-surgical plans.

## Data Availability

The original contributions presented in the study are included in the article, further inquiries can be directed to the corresponding author.
